# Supplementary material for: Macaque dorsal premotor cortex exhibits decision-related activity only when specific stimulus–response associations are known
Source: Nat Commun. 2019 Apr 17;10:1793. doi: 10.1038/s41467-019-09460-y (PMC6470163; doi:10.1038/s41467-019-09460-y)
Supplement: Supplementary file 1 — Supplementary Information [file 41467_2019_9460_MOESM1_ESM.pdf]

Title

Macaque dorsal premotor cortex exhibits decision-related activity only when specific stimulus-response associations are known

Authors/Affiliations

Megan Wang[1,*], Christéva Montanède[2,*], Chandramouli Chandrasekaran[3,4,5,6], Diogo Peixoto[7,8], Krishna V. Shenoy[3,4,7,9,10,11], John F. Kalaska[2]

[1]Neurosciences Graduate Program, Stanford University, Stanford, CA 94305, USA.
[2]Département de Neurosciences, Pavillon Paul-G.-Desmarais, Faculté de Médecine, Université de Montréal, succursale Centre-ville, Montréal, Québec, H3C 3J7, Canada.
[3]Department of Electrical Engineering, [4]Howard Hughes Medical Institute, [5]Department of Anatomy and Neurobiology, Boston University, Boston, MA, 02118, [6]Department of Psychological and Brain Sciences, Boston University, Boston, MA, 02118, [7]Department of Neurobiology, Stanford University, Stanford, CA 94305, USA. [8]Champalimaud Neuroscience Programme, Lisbon 1400-038, Portugal. [9]Department of Bioengineering, [10]Bio-X Program, [11]Stanford Neurosciences Institute, Stanford University, Stanford, CA 94305, USA.
*These first authors contributed equally.
Correspondence should be addressed to J.F.K. (john.francis.kalaska@umontreal.ca)

Supplementary Figures

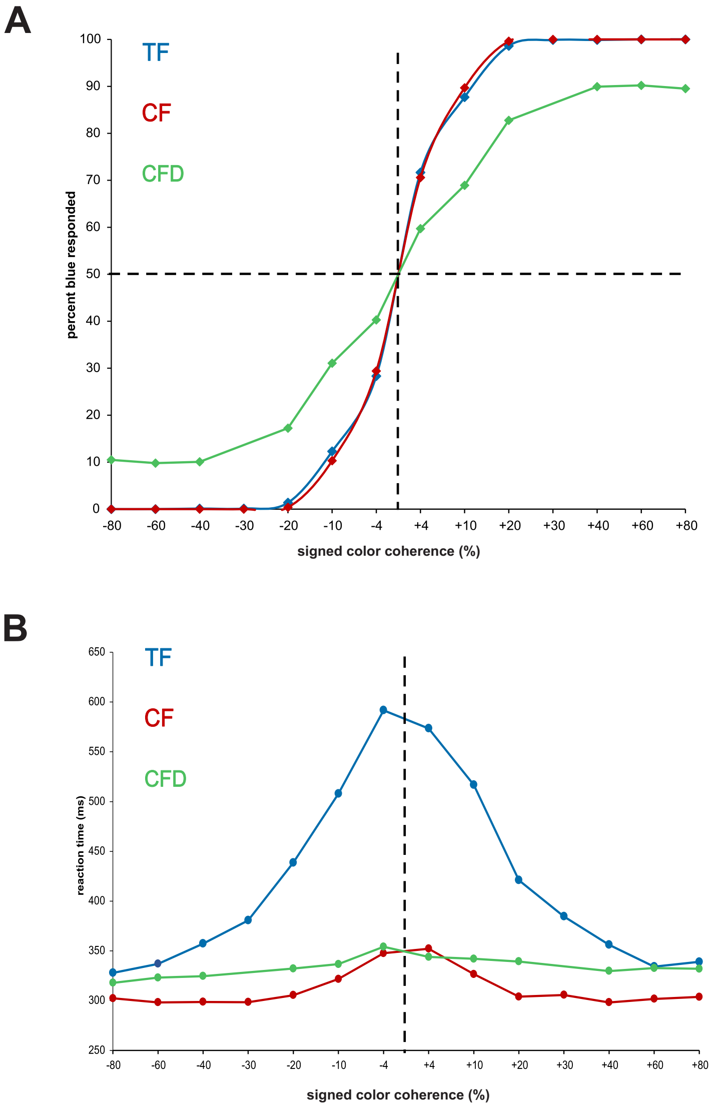

Supplementary Figure 1. Monkey Z behavior for all tasks: TF, CF, and CFD. **A)** Psychometric curves and **B)** chronometric curves are shown; compare to Figure 1 **B,C**). TF and CF tasks: same data as in Figure 1B,C. **A)** Like monkey T, monkey Z showed a decrease in success rates for the checkerboards with the highest color coherences in the CFD task compared to the TF and CF tasks. **B)** Unlike monkey T, however, monkey Z continued to show a substantial reduction in RTs for checkerboards with lower color coherences in the CFD task (green line) compared to the TF task (blue line).

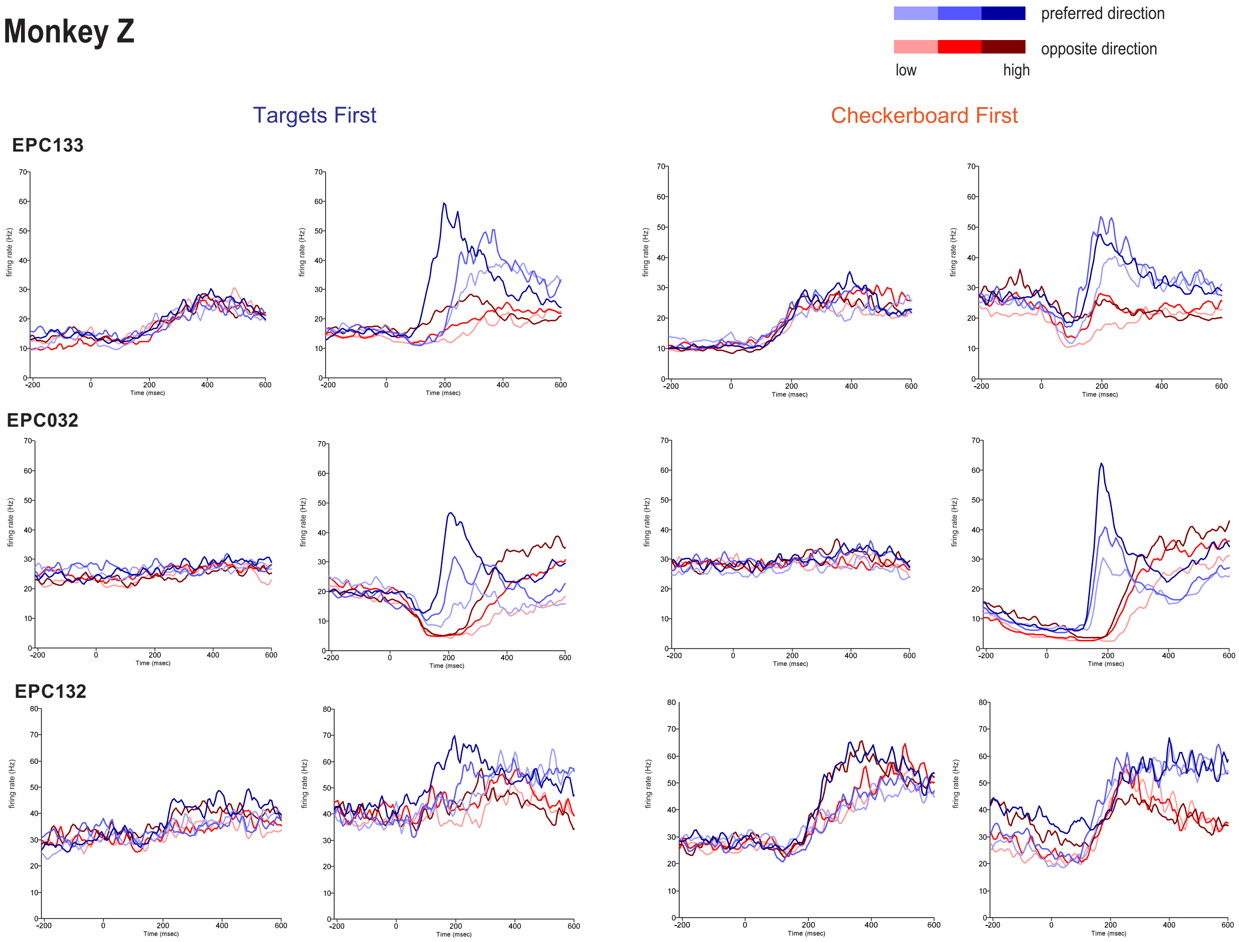

Supplementary Figure 2. Further examples of units with responses to first visual cue onset; same format as Figure 3. Unit EPC133 (top) showed a significant rapid response onset (see Methods) at 220ms after the Targets cue appeared in the TF task and 180ms after the Checkerboard appeared in the CF task. Unit EPC032 (middle) did not show a significant rapid response to the Targets in the TF task but showed a late rapid decrease in activity (>600ms) to the Checkerboard in the CF task. Unit EPC132 (bottom) showed a significant rapid increase in response to the Targets at 280ms after they appeared in the TF task, and a significant rapid response increase 220ms after the Checkerboard appeared in the CF task. The response to the Checkerboard was markedly stronger and more rapid for the 100% coherence than for the 20% and 4% coherences. EPC132 showed a significant main effect of evidence Strength in the ANOVA, but did not show a significant linear regression to either the signed color coherence or the signed evidence for reach direction during the Checkerboard-observation period of the CF task.

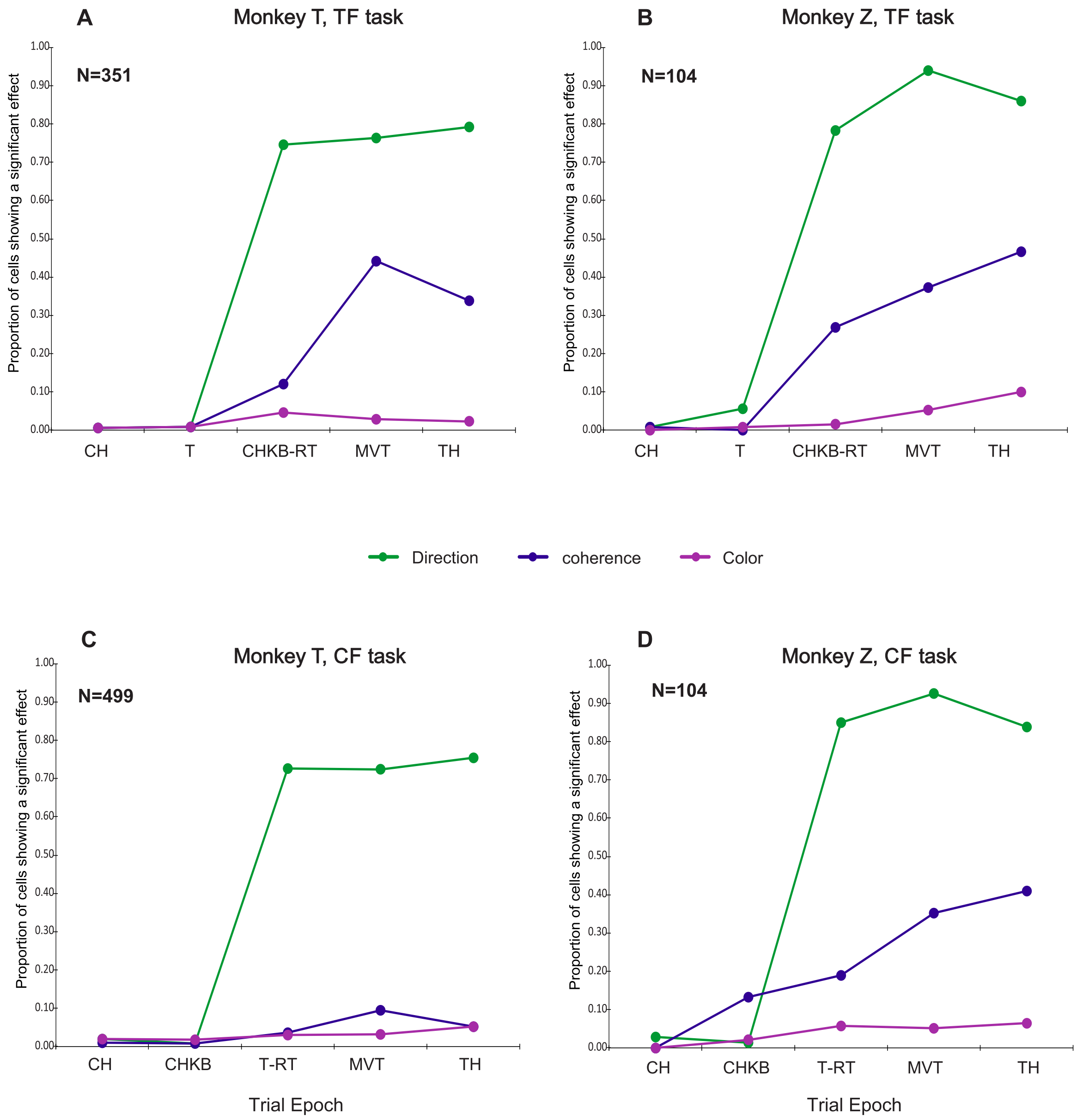

Supplementary Figure 3. Proportion of the units that showed a significant main effect of the task factors chosen reach Direction, unsigned checkerboard evidence Strength and checkerboard dominant Color in different trial epochs (ANOVA, p< 0.01). **A,B**) TF task. Very few units showed a significant main effect of any task factors during the Center-hold (CH) and Targets-observation (T) epochs. Furthermore, very few units in either monkey showed a significant modulation by the color-location conjunction information provided by the Targets cue during the Targets-observation period (Direction X Color interaction, $p < 0.01$; Supplemental Table 1). After the checkerboard appeared, many units in both monkeys showed a significant effect of Direction (green) during the Checkerboard-RT epoch before the onset of the reaching movements (CHKB-RT), during the Movement epoch toward the targets (MVT) and during the

Target-Hold epoch at the end of the reaching movements (TH). A smaller proportion of units showed a significant main effect of checkerboard evidence Strength (blue) during the CHKB-RT, MVT and TH epochs. Very few units in either monkey showed a significant effect of checkerboard dominant Color (magenta) in any trial epoch. Many units in both monkeys also showed a significant effect of evidence Strength on their Direction responses (significant Direction X Strength interaction, $p < 0.01$). The effects of evidence Strength and the Direction X Strength interaction captured most of the effect of checkerboard coherence on the directional activity of the units in the TF task in all trial epochs after the checkerboard appeared (Figure 2, 3; Supplemental Figure 2). **C,D**) CFD/CF task. Very few units in either monkey showed a significant main effect of Direction during the CH and Checkerboard-observation (CHKB) epochs. Many units emitted strongly Direction signals during the Targets-RT (T-RT), MVT and TH epochs. In monkey T (**C**), relatively few units showed a significant main effect of checkerboard evidence Strength. In contrast, some units in monkey Z (**D**) were significantly modulated by checkerboard Strength during the CHBK epoch, and the number of significant effects of Strength increased progressively during the T-RT, MVT and TH epochs, but fewer than in the TF task. Very few units in either monkey showed a significant effect of checkerboard dominant Color, or Color X Direction or Color X Strength interactions in any trial epoch (see Supplemental Table 1).

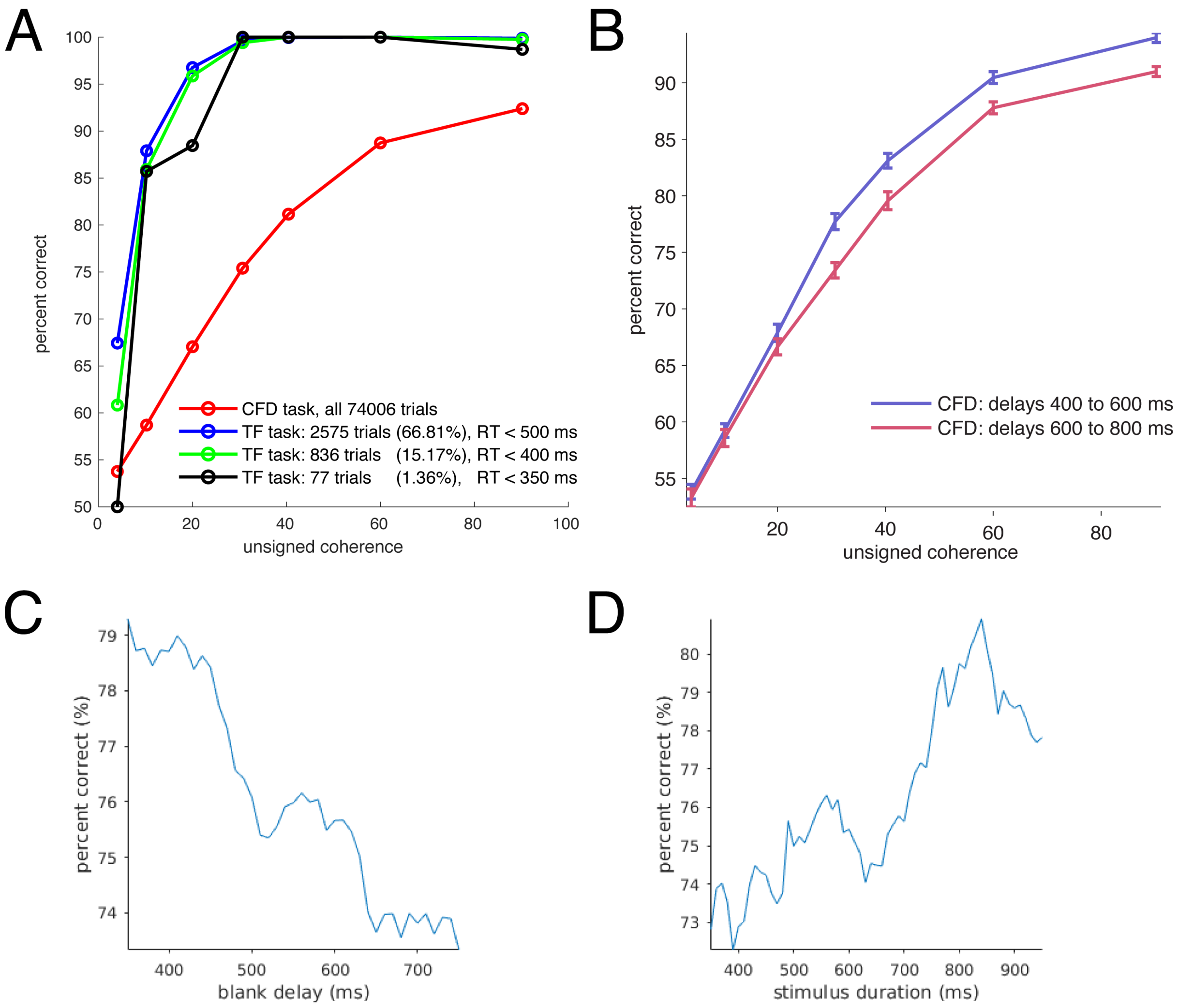

Supplementary Figure 4. Monkey T's behavior as a function of checkerboard duration and blank delay duration.

A) We can compare the performance in the CFD task with performance in the TF task with trials conditioned on reaction time. Since the checkerboard disappears once the hand begins to move, reaction times less than 500 ms mean that the checkerboard viewing time was 500 ms or less in the TF task. Even for reaction times 350 ms or less, the performance is much better than in the CFD task. Thus, the 500 ms checkerboard viewing time in CFD is sufficient to achieve very reasonable discrimination performance.

B) Within all CFD trials, sorting by shorter (400 to 600 ms) and longer (600 to 800 ms) delays reveals that performance is better for shorter delays with a slight sharpening of the slope of the psychometric curve as well as lower lapse rates. Data points are means ± standard error over sessions.

C,D) To further assess the effect of timing on performance, a subset of 9 sessions explored blank delay durations ranging 300 to 800 ms, and checkerboard stimulus durations ranging 300 to 1000 ms. The percent correct (across all coherences)

increases for shorter delays and longer checkerboard durations. These percent correct were calculated in 100 ms bins (e.g. the percent correct for blank delays 300 to 400 ms is plotted at 350 ms) stepping over the range in 10 ms time steps (e.g. the next data point, plotted at 360 ms, calculates percent correct for blank delays 310 to 410 ms).

Supplementary Tables

A. Targets First task

Factor		Monkey T (N = 351 units)						Monkey Z (N = 104 units)					
Factor		D	C	S	DxC	DxS	CxS	D	C	S	DxC	DxS	CxS
Center-hold	Units	2	2	2	8	2	3	1	0	1	0	0	2
	Fraction	0.01	0.01	0.01	0.02	0.01	0.01	0.01	0.00	0.01	0.00	0.00	0.02
Targets	Units	3	3	3	1	2	0	5	1	0	5	1	1
	Fraction	0.01	0.01	0.01	0.00	0.01	0.00	0.05	0.01	0.00	0.05	0.01	0.01
Checkerboard-RT	Units	260	16	42	7	16	2	79	2	26	5	10	1
	Fraction	0.74	0.05	0.12	0.02	0.05	0.01	0.76	0.02	0.25	0.05	0.10	0.01
Movement	Units	266	10	154	6	61	5	95	7	39	4	19	2
	Fraction	0.76	0.03	0.44	0.02	0.17	0.01	0.91	0.07	0.38	0.04	0.18	0.02
Target-hold	Units	276	8	118	3	57	1	86	10	49	2	20	2
	Fraction	0.79	0.02	0.34	0.01	0.16	0.00	0.83	0.1	0.47	0.02	0.19	0.02

B. Checkerboard First with Delay / Checkerboard First

Factor		Monkey T (N = 499 units)						Monkey Z (N = 104 units)					
Factor		D	C	S	DxC	DxS	CxS	D	C	S	DxC	DxS	CxS
Center-hold	Units	10	10	5	1	5	3	3	0	0	0	2	2
	Fraction	0.02	0.02	0.01	0.00	0.01	0.01	0.03	0.00	0.00	0.00	0.02	0.02
Checkerboard	Units	4	9	4	4	1	5	1	2	12	0	1	0
	Fraction	0.01	0.02	0.01	0.01	0.00	0.01	0.01	0.02	0.12	0.00	0.01	0.00
Targets-RT	Units	360	15	18	17	3	1	88	6	22	4	8	0
	Fraction	0.72	0.03	0.04	0.03	0.01	0.00	0.85	0.06	0.21	0.04	0.08	0.00
Movement	Units	359	16	47	5	15	2	94	6	38	4	18	1
	Fraction	0.72	0.03	0.09	0.01	0.03	0.00	0.90	0.06	0.37	0.04	0.17	0.01
Target-hold	Units	374	26	26	14	17	4	84	7	44	5	15	2
	Fraction	0.75	0.05	0.05	0.03	0.03	0.01	0.81	0.07	0.42	0.05	0.14	0.02

Supplementary Table 1. ANOVA results for monkey T and monkey Z for both tasks: Targets First (A) and Checkerboard First with Delay / Checkerboard First (B); see Supplementary Figure 3. D, chosen reach direction; C, checkerboard dominant color; S, unsigned checkerboard coherence strength; DxC, DxS, CxS, interactions between variables.

Supplementary Discussion

PMd units in monkey Z but not monkey T exhibited responses to the first sensory information provided in each task

The response of many PMd units in monkey Z to the appearance of the Target cues in the TF task is consistent with a similar activation of PMd units in a 2-Target memorized-delay task. That activity after the presentation of the two colored target cues has been interpreted as a simultaneous co-activation of two PMd populations that prefer reaches to the two potential targets before a second monochromatic instructional cue identifies the correct target[1,2]. This activity is sustained during the post-Targets memory-delay period of the 2-Target task, and is a putative neural correlate of a memorized representation of the Target cue information in each trial, expressed in the space of potential actions in PMd (c.f., Horwitz et al[3]).

The responses of the units to the appearance of the Checkerboard cues in the CF task likely do not implicate PMd in the perceptual interpretation of the color evidence since the responses were largely insensitive to the salient dimensions (dominant color, signed color coherence) of the stimuli (Figure 5, 6). Alternatively, the activation may reflect a similar process of representation of potential actions as in the TF task. Once a unit in monkey Z was chosen for recording in a given recording session, it was tested with the same two opposite target locations in the task-defined preferred reach direction of the unit and the opposite direction for hundreds of trials in the two tasks. Therefore, it is reasonable to expect that monkey Z had some level of implicit prior knowledge of where the two targets would appear at the start of each trial in both tasks and all that it lacked was the specific color-location conjunction, which it obtained when the targets appeared. The activity during the Checkerboard observation period in the CF task may have reflected the accumulating knowledge of the dominant color of the checkerboard in neural circuits outside of PMd. This may have enabled a covert activation of the simultaneous representations of the two anticipated potential actions even before the targets actually appeared in the CF task since that accumulating sensory evidence will eventually support one or the other of the two colored targets once they appeared. This coactivation of the two PMd populations preferring the two targets may have in turn contributed to the shorter RTs in the CF task than the TF task for even the 100% checkerboards. Critically, however, the linear regression (Figure 5), ROC (Figure 6) and ANOVA (Supplemental Figure 3) showed that these activations during the first-cue observation periods in PMd of monkey Z did not reflect the final decision-related processes as defined here because it did not predict any aspect of monkey Z's differential choice behavior after the second visual cue appeared in each task.

Since activation of PMd by partial action-related information before the final action choice can be specified is a robust finding in several different studies[1,2,4-15], why did monkey T not show any activity in PMd during the first visual-cue observation periods in the TF and CFD tasks? This could occur if different cortical regions were sampled in

monkeys T and Z. Both monkeys are still participating in neural recordings and histological localization of penetration sites has not yet been done. However, similar stereotactic coordinates for chamber implants and extensive relative overlap of recording penetrations within the chambers in the two monkeys suggest that is not the main cause (Figure 1E).

Another possible explanation is the different training history of the two monkeys. Monkey T only ever experienced L/R targets and was initially trained in the TF task in which the targets remained visible for the duration of each trial. Thus, its original training experience did not require the establishment of a memorized trace of the target information. In contrast, monkey Z was trained for many months in the 1T and 2T tasks with targets in 8 different directions that varied from trial to trial and with two long sequential memory-delay periods during which the monkey had to remember the spatial location (1T task) and color-location conjunctions of the targets in each trial before selecting a target (2T task), and initiating a reach (1T and 2T tasks). The 1T and 2T tasks were also used in all neural recording sessions to search for task-related units. Engagement of PMd during the memory-delay periods of the 1T and 2T tasks may have facilitated task performance for monkey Z, which was carried over to the TF and CF tasks.

Another possible contributing factor is the target location placement in the tasks. For monkey Z, targets were placed in spatial locations along the preferred-opposite movement direction axis of each unit to maximize the difference in their directional activity in the TF and CF tasks, and would change from unit to unit. For monkey T, in contrast, target locations were fixed to the left and right of center, and units were recorded for that single movement axis, regardless of what their preferred reach directions might have been.

Finally, the lack versus presence of activity changes after the checkerboards appeared in the CF/CFD tasks may have reflected a difference in the strategy that the two monkeys adopted to perform the tasks. Monkey T may have adopted a strategy whereby it attempted to store a purely “sensory” mnemonic representation of the checkerboard stimuli but largely deferred the interpretation of the checkerboard evidence until the appearance of the targets. This resulted in no PMd responses and longer RTs in the CFD task compared to the TF task. In contrast, monkey Z appeared to largely commit to a categorical decision about the dominant color of the checkerboard while observing it in the CF task, resulting in a substantial reduction in RTs after the targets appeared. This may have been accompanied by a covert activation of the two competing action-related PMd populations, like in the TF task, while monkey Z deliberated on the color evidence in the checkerboard, even though the targets had not yet appeared. This is further reinforced by the finding that monkey Z’s RTs remained drastically shorter in a modified version of the CF task that had the same temporal structure as the CFD task. This showed that monkey T’s prolonged RTs in the CFD task compared to the TF task were not due solely to the imposed memory-delay period but

rather to how and when it interpreted that task-relevant sensory evidence provided by the checkerboards.

How did the monkeys convert checkerboard color coherence into reach actions?

The systematic differences in the rate of rise of reach-related directional signals in PMd as a function of the color coherence of the checkerboards in the TF task are reminiscent of similar correlations with the coherent motion strength of RDK stimuli seen in saccade-related cortical regions[16-18] and in parietal cortex area 5 in an arm reach task[19]. Those findings have been interpreted as the neural correlate of a process of accumulation of noisy sensory evidence across time using signals generated by motion-sensitive neurons in medial temporal cortex (MT) to inform the choice of the action that must be performed to report a decision about perceived net motion direction. This makes intuitive sense since the RDK stimuli are stochastic, contain variable amounts of dot motion in random directions as well as in the coherent motion direction from moment to moment, and only evoke reliable sensations of coherent visual motion in a particular direction when experienced over time[17].

In contrast, the checkerboards used in these tasks comprised sets of small squares whose colors are easily and rapidly discriminable, and contained no input signal “noise” comparable to the variable numbers of dots moving in the coherent and random directions from frame to frame in the RDK stimuli. The checkerboard stimuli presented in half of the trials to monkey Z were dynamic and changed every 50ms, but the numbers of blue and yellow squares in each stimulus stream remained fixed in a given trial and only their positions within the checkerboard changed from image frame to image frame. In contrast, in the other half of the trials for monkey Z and all of the trials for monkey T, a single static checkerboard of R/G or B/Y squares appeared for the duration of the observation period in each trial, so that the physical properties of the sensory stimulus that the monkeys experienced did not change across time. Despite these differences in the visual stimuli, the two monkeys showed remarkably similar chronometric and psychophysical trends in the TF task (present study; [20]). Furthermore, human and non-human subjects also showed very similar performance when viewing either dynamic or static checkerboard stimuli ([2,21]; present study). Thus, whereas the motion sensations evoked by RDK stimuli require dynamically changing stimuli across time, the assessment of the color evidence in the checkerboards was relatively insensitive to the presence or absence of continually updated sensory inputs. This does not, however, preclude momentary stochastic noise generated within the central neural circuits that process even the static checkerboard visual input (both monkeys) and store it in short-term working memory (monkey T).

The similarity of task performance in the TF task is also striking given another difference in the checkerboard stimuli experienced by the two monkeys. The checkerboards used in monkey T’s experiments contained only task-salient R and G squares. In contrast, the checkerboards used in monkey Z’s experiments contained 100 task-salient B and Y

squares against a background of 125 task-irrelevant R squares. This reduced the overall density of task-relevant color information in the checkerboards for monkey Z and required it to identify the task-relevant information from among the “distractor” red squares. Despite this difference, the psychophysical curves and psychophysical thresholds of the two monkeys were very similar in the TF task, and the RTs for the high-coherence checkerboards were actually shorter in monkey Z than monkey T (Figure 1).

Despite the absence of color evidence “noise” in the checkerboards and the insensitivity of task performance to static versus dynamic stimuli, the monkeys took longer to choose a colored target when the checkerboard coherence decreased[2,20,21]. In RDK stimuli, this effect has been explained by a sequential-sampling process that takes longer to identify the direction of the weak coherent-motion signal generated by MT neurons against a high level of motion direction noise. For the checkerboard stimuli, in contrast, it presumably reflects a longer period of time required to determine whether the checkerboard was predominantly one or the other of the two task-salient colors as the numbers of squares of the two easily-discriminable colors became more similar. This may require longer re-sampling of the sensory input while observing the checkerboard (monkey Z) or from a noisy working-memory trace of the checkerboard (monkey T; [22,23], Shushruth and Shadlen, CoSyNe abstract). We can assume that the color evidence is initially processed by neurons in the parvocellular “color-opponent” pathway[24-27]. However, to our knowledge, there have been no studies of neural responses in that pathway to multi-colored checkerboard stimuli like ours in color discrimination tasks analogous to the many studies of visual motion processing in MT.

The perceptual decision could be considered as a pure color discrimination problem since the subjects had to estimate the dominant color of the checkerboards in order to identify the reach target whose color matched that of the checkerboard. However, similar dichromatic dot arrays have been used in studies of numerosity, the ability of subjects to estimate relative numbers of visual objects[28-30]. Subjects likewise showed longer RTs when the relative numbers of objects in the stimuli are similar[30,31]. These results have been interpreted as consistent with a process of sequential sampling and accumulation of evidence across time and across space within the stimuli[30-33], but did not speculate on the nature of the sensory evidence that was being sampled, unlike RDK stimuli. Furthermore, the checkerboards that we used have inherent in them several potential confounding “low level” physical properties identified in numerosity studies that are independent of the presumably “higher level” sense of relative numbers per se, including the relative area of the checkerboard occupied by squares of each color, their total circumference, and the relative degree of spatial contiguity of squares of the same color (i.e., how often they cluster to share a common border)[30,34-37]. Indeed, the colored squares did not have a neutral-colored border and so would form larger monochromatic “clumps” when contiguous, rather than remaining visible as discrete squares (Figure 1D). All of these factors could have contributed to the monkeys' estimation of the dominant color of the checkerboards, independent of any estimate of

relative numbers of squares. Furthermore, the number and density of squares in our checkerboards were usually higher than normally used in numerosity studies and more closely resemble what are called “textures”, which follow different psychophysical laws than dot arrays with smaller numbers of elements[28].

However, this study was not designed to study numerosity or to examine what specific properties of the checkerboards the subjects used to make the relative color estimates. Instead, the checkerboards were chosen as a means to present stimuli with different levels of competing evidence for two alternative reach choices, using a stimulus dimension (color) that has no inherent natural association with the directionality of motor output. Our findings indicate that PMd units express activity pertaining to the likelihood of different action choices provided by the checkerboard stimuli, independent of the critical decision-relevant physical property of the sensory input on which those action likelihoods are based, in this case its dominant color.

Important questions not directly addressed by this study are where are the neural correlates of the critical color-related information on which the action decisions were based and how are they transformed into color-independent evidence supporting the action choices? A strong candidate is the dorsolateral prefrontal cortex[38,39]. We have preliminary evidence that the specific color/location conjunctions of the spatial target cues and color/location matching rules after the checkerboard appeared in each trial are expressed in lateral prefrontal cortex around the principal sulcus while a monkey performed a TF task (Coallier et al., 2008, SfN abstract).

The effect of checkerboard color coherence on task performance and PMd neural activity is consistent with a number of different computational decision-making models, including drift-diffusion[17,18,30,40,41]; gated stochastic accumulation[42,43], urgency gating[44,45], and independent-race[46-48]. Nevertheless, we acknowledge that our neural data are correlational and are not proof of a causal relationship between PMd activity and either perceptual or motor decisions. Furthermore, until more neurophysiological findings are available about the sources and nature of sensory signals that are being processed while subjects estimate the relative amounts of colored squares in the dichromatic checkerboard stimuli, and how those sensory signals are transformed into action-related information, we prefer to remain agnostic as to the computational mechanisms that underlie the task performance of the subjects.

Supplementary References

1 Cisek, P. & Kalaska, J. F. Neural correlates of reaching decisions in dorsal premotor cortex: specification of multiple direction choices and final selection of action. *Neuron* **45**, 801-814, doi:10.1016/j.neuron.2005.01.027 (2005).

2 Coallier, E., Michelet, T. & Kalaska, J. F. Dorsal premotor cortex: neural correlates of reach target decisions based on a color-location matching rule and conflicting sensory evidence. *J Neurophysiol* **113**, 3543-3573, doi:10.1152/jn.00166.2014 (2015).

3 Horwitz, G. D., Batista, A. P. & Newsome, W. T. Representation of an abstract perceptual decision in macaque superior colliculus. *J Neurophysiol* **91**, 2281-2296, doi:10.1152/jn.00872.2003 (2004).

4 Cisek, P. & Kalaska, J. F. Simultaneous encoding of multiple potential reach directions in dorsal premotor cortex. *J Neurophysiol* **87**, 1149-1154 (2002).

5 di Pellegrino, G. & Wise, S. P. Visuospatial versus visuomotor activity in the premotor and prefrontal cortex of a primate. *J Neurosci* **13**, 1227-1243 (1993).

6 Hoshi, E. & Tanji, J. Integration of target and body-part information in the premotor cortex when planning action. *Nature* **408**, 466-470, doi:10.1038/35044075 (2000).

7 Hoshi, E. & Tanji, J. Differential involvement of neurons in the dorsal and ventral premotor cortex during processing of visual signals for action planning. *J Neurophysiol* **95**, 3596-3616, doi:10.1152/jn.01126.2005 (2006).

8 Nakayama, Y., Yamagata, T., Tanji, J. & Hoshi, E. Transformation of a virtual action plan into a motor plan in the premotor cortex. *J Neurosci* **28**, 10287-10297, doi:10.1523/JNEUROSCI.2372-08.2008 (2008).

9 Riehle, A. & Requin, J. Monkey primary motor and premotor cortex: single-cell activity related to prior information about direction and extent of an intended movement. *J Neurophysiol* **61**, 534-549 (1989).

10 Rossi-Pool, R. *et al.* Emergence of an abstract categorical code enabling the discrimination of temporally structured tactile stimuli. *Proc Natl Acad Sci U S A* **113**, E7966-E7975, doi:10.1073/pnas.1618196113 (2016).

11 Rossi-Pool, R. *et al.* Decoding a Decision Process in the Neuronal Population of Dorsal Premotor Cortex. *Neuron* **96**, 1432-1446 e1437, doi:10.1016/j.neuron.2017.11.023 (2017).

12 Wallis, J. D. & Miller, E. K. From rule to response: neuronal processes in the premotor and prefrontal cortex. *J Neurophysiol* **90**, 1790-1806, doi:10.1152/jn.00086.2003 (2003).

13 Wise, S. P., Di Pellegrino, G. & Boussaoud, D. Primate premotor cortex: dissociation of visuomotor from sensory signals. *J Neurophysiol* **68**, 969-972 (1992).

14 Yamagata, T., Nakayama, Y., Tanji, J. & Hoshi, E. Processing of visual signals for direct specification of motor targets and for conceptual representation of action targets in the dorsal and ventral premotor cortex. *J Neurophysiol* **102**, 3280-3294, doi:10.1152/jn.00452.2009 (2009).

15 Yamagata, T., Nakayama, Y., Tanji, J. & Hoshi, E. Distinct information representation and processing for goal-directed behavior in the dorsolateral and ventrolateral prefrontal cortex and the dorsal premotor cortex. *J Neurosci* **32**, 12934-12949, doi:10.1523/JNEUROSCI.2398-12.2012 (2012).
16 Kim, J. N. & Shadlen, M. N. Neural correlates of a decision in the dorsolateral prefrontal cortex of the macaque. *Nat Neurosci* **2**, 176-185, doi:10.1038/5739 (1999).
17 Roitman, J. D. & Shadlen, M. N. Response of neurons in the lateral intraparietal area during a combined visual discrimination reaction time task. *J Neurosci* **22**, 9475-9489 (2002).
18 Shadlen, M. N. & Newsome, W. T. Neural basis of a perceptual decision in the parietal cortex (area LIP) of the rhesus monkey. *J Neurophysiol* **86**, 1916-1936 (2001).
19 de Lafuente, V., Jazayeri, M. & Shadlen, M. N. Representation of accumulating evidence for a decision in two parietal areas. *J Neurosci* **35**, 4306-4318, doi:10.1523/JNEUROSCI.2451-14.2015 (2015).
20 Chandrasekaran, C., Peixoto, D., Newsome, W. T. & Shenoy, K. V. Laminar differences in decision-related neural activity in dorsal premotor cortex. *Nat Commun* **8**, 614, doi:10.1038/s41467-017-00715-0 (2017).
21 Coallier, E. & Kalaska, J. F. Reach target selection in humans using ambiguous decision cues containing variable amounts of conflicting sensory evidence supporting each target choice. *J Neurophysiol* **112**, 2916-2938, doi:10.1152/jn.00145.2014 (2014).
22 Pearson, B., Raskevicius, J., Bays, P. M., Pertzov, Y. & Husain, M. Working memory retrieval as a decision process. *J Vis* **14**, doi:10.1167/14.2.2 (2014).
23 Shadlen, M. N. & Shohamy, D. Decision Making and Sequential Sampling from Memory. *Neuron* **90**, 927-939, doi:10.1016/j.neuron.2016.04.036 (2016).
24 Bohon, K. S., Hermann, K. L., Hansen, T. & Conway, B. R. Representation of Perceptual Color Space in Macaque Posterior Inferior Temporal Cortex (the V4 Complex). *eNeuro* **3**, doi:10.1523/ENEURO.0039-16.2016 (2016).
25 Cheadle, S. W. & Zeki, S. The role of parietal cortex in the formation of color and motion based concepts. *Front Hum Neurosci* **8**, 535, doi:10.3389/fnhum.2014.00535 (2014).
26 Conway, B. R. Color signals through dorsal and ventral visual pathways. *Vis Neurosci* **31**, 197-209, doi:10.1017/S0952523813000382 (2014).
27 Conway, B. R. & Livingstone, M. S. Spatial and temporal properties of cone signals in alert macaque primary visual cortex. *J Neurosci* **26**, 10826-10846, doi:10.1523/JNEUROSCI.2091-06.2006 (2006).
28 Burr, D. C., Anobile, G. & Arrighi, R. Psychophysical evidence for the number sense. *Philos Trans R Soc Lond B Biol Sci* **373**, doi:10.1098/rstb.2017.0045 (2017).
29 Cantlon, J. F., Platt, M. L. & Brannon, E. M. Beyond the number domain. *Trends Cogn Sci* **13**, 83-91, doi:10.1016/j.tics.2008.11.007 (2009).

30 Ratcliff, R. & McKoon, G. Modeling numerosity representation with an integrated diffusion model. *Psychol Rev* **125**, 183-217, doi:10.1037/rev0000085 (2018).

31 Ratcliff, R., Thompson, C. A. & McKoon, G. Modeling individual differences in response time and accuracy in numeracy. *Cognition* **137**, 115-136, doi:10.1016/j.cognition.2014.12.004 (2015).

32 Ratcliff, R. Measuring psychometric functions with the diffusion model. *J Exp Psychol Hum Percept Perform* **40**, 870-888, doi:10.1037/a0034954 (2014).

33 Fornaciai, M. & Park, J. Spatiotemporal feature integration shapes approximate numerical processing. *J Vis* **17**, 6, doi:10.1167/17.13.6 (2017).

34 Gebuis, T. & Reynvoet, B. The interplay between nonsymbolic number and its continuous visual properties. *J Exp Psychol Gen* **141**, 642-648, doi:10.1037/a0026218 (2012).

35 Dietrich, J. F., Huber, S. & Nuerk, H. C. Methodological aspects to be considered when measuring the approximate number system (ANS) - a research review. *Front Psychol* **6**, 295, doi:10.3389/fpsyg.2015.00295 (2015).

36 Harvey, B. M. & Dumoulin, S. O. Can responses to basic non-numerical visual features explain neural numerosity responses? *Neuroimage* **149**, 200-209, doi:10.1016/j.neuroimage.2017.02.012 (2017).

37 Leibovich, T. & Henik, A. Magnitude processing in non-symbolic stimuli. *Front Psychol* **4**, 375, doi:10.3389/fpsyg.2013.00375 (2013).

38 di Pellegrino, G. & Wise, S. P. A neurophysiological comparison of three distinct regions of the primate frontal lobe. *Brain* **114 (Pt 2)**, 951-978 (1991).

39 Mante, V., Sussillo, D., Shenoy, K. V. & Newsome, W. T. Context-dependent computation by recurrent dynamics in prefrontal cortex. *Nature* **503**, 78-84, doi:10.1038/nature12742 (2013).

40 Gold, J. I. & Shadlen, M. N. The neural basis of decision making. *Annu Rev Neurosci* **30**, 535-574, doi:10.1146/annurev.neuro.29.051605.113038 (2007).

41 Ratcliff, R., Smith, P. L., Brown, S. D. & McKoon, G. Diffusion Decision Model: Current Issues and History. *Trends Cogn Sci* **20**, 260-281, doi:10.1016/j.tics.2016.01.007 (2016).

42 Purcell, B. A., Schall, J. D., Logan, G. D. & Palmeri, T. J. From salience to saccades: multiple-alternative gated stochastic accumulator model of visual search. *J Neurosci* **32**, 3433-3446, doi:10.1523/JNEUROSCI.4622-11.2012 (2012).

43 Schall, J. D., Purcell, B. A., Heitz, R. P., Logan, G. D. & Palmeri, T. J. Neural mechanisms of saccade target selection: gated accumulator model of the visual-motor cascade. *Eur J Neurosci* **33**, 1991-2002, doi:10.1111/j.1460-9568.2011.07715.x (2011).

44 Cisek, P., Puskas, G. A. & El-Murr, S. Decisions in changing conditions: the urgency-gating model. *J Neurosci* **29**, 11560-11571, doi:10.1523/JNEUROSCI.1844-09.2009 (2009).

45 Thura, D. & Cisek, P. Deliberation and commitment in the premotor and primary motor cortex during dynamic decision making. *Neuron* **81**, 1401-1416, doi:10.1016/j.neuron.2014.01.031 (2014).

46 Brown, S. D. & Heathcote, A. The simplest complete model of choice response time: linear ballistic accumulation. *Cogn Psychol* **57**, 153-178, doi:10.1016/j.cogpsych.2007.12.002 (2008).

47 Carpenter, R. H. & Williams, M. L. Neural computation of log likelihood in control of saccadic eye movements. *Nature* **377**, 59-62, doi:10.1038/377059a0 (1995).

48 Noorani, I. & Carpenter, R. H. The LATER model of reaction time and decision. *Neurosci Biobehav Rev* **64**, 229-251, doi:10.1016/j.neubiorev.2016.02.018 (2016).
