# Supplementary material for: Macaque dorsal premotor cortex exhibits decision-related activity only when specific stimulus–response associations are known
Source: Nat Commun. 2019 Apr 17;10:1793. doi: 10.1038/s41467-019-09460-y (PMC6470163; doi:10.1038/s41467-019-09460-y)
Supplement: Supplementary file 3 — Reporting Summary [file 41467_2019_9460_MOESM3_ESM.pdf]

natureresearch

Corresponding author(s): John Kalaska

Reporting Summary

Nature Research wishes to improve the reproducibility of the work that we publish. This form provides structure for consistency and transparency in reporting. For further information on Nature Research policies, see Authors & Referees and the Editorial Policy Checklist.

Statistical parameters

When statistical analyses are reported, confirm that the following items are present in the relevant location (e.g. figure legend, table legend, main text, or Methods section).

n/a	Confirmed	
☐	☒	The exact sample size (*n*) for each experimental group/condition, given as a discrete number and unit of measurement
☐	☒	An indication of whether measurements were taken from distinct samples or whether the same sample was measured repeatedly
☐	☒	The statistical test(s) used AND whether they are one- or two-sided<br>*Only common tests should be described solely by name; describe more complex techniques in the Methods section.*
☐	☒	A description of all covariates tested
☐	☒	A description of any assumptions or corrections, such as tests of normality and adjustment for multiple comparisons
☐	☒	A full description of the statistics including central tendency (e.g. means) or other basic estimates (e.g. regression coefficient) AND variation (e.g. standard deviation) or associated estimates of uncertainty (e.g. confidence intervals)
☐	☒	For null hypothesis testing, the test statistic (e.g. *F*, *t*, *r*) with confidence intervals, effect sizes, degrees of freedom and *P* value noted<br>*Give P values as exact values whenever suitable.*
☒	☐	For Bayesian analysis, information on the choice of priors and Markov chain Monte Carlo settings
☒	☐	For hierarchical and complex designs, identification of the appropriate level for tests and full reporting of outcomes
☒	☐	Estimates of effect sizes (e.g. Cohen's *d*, Pearson's *r*), indicating how they were calculated
☐	☒	Clearly defined error bars<br>*State explicitly what error bars represent (e.g. SD, SE, CI)*

Our web collection on statistics for biologists may be useful.

Software and code

Policy information about availability of computer code

Data collection	Custom code were used to collect data in this study, as well as commercial code (MATLAB) and open source code (toolboxes created for MATLAB such as PsychToolBoX).
Data analysis	Custom code were used to analyze data in this study, as well as commercial code part of the MATLAB language..

For manuscripts utilizing custom algorithms or software that are central to the research but not yet described in published literature, software must be made available to editors/reviewers upon request. We strongly encourage code deposition in a community repository (e.g. GitHub). See the Nature Research guidelines for submitting code & software for further information.

Data

Policy information about availability of data

All manuscripts must include a data availability statement. This statement should provide the following information, where applicable:

- Accession codes, unique identifiers, or web links for publicly available datasets
- A list of figures that have associated raw data
- A description of any restrictions on data availability

The data that support the findings of this study are available from the corresponding author upon reasonable request.

Field-specific reporting

Please select the best fit for your research. If you are not sure, read the appropriate sections before making your selection.

☒ Life sciences ☐ Behavioural & social sciences ☐ Ecological, evolutionary & environmental sciences

For a reference copy of the document with all sections, see nature.com/authors/policies/ReportingSummary-flat.pdf

Life sciences study design

All studies must disclose on these points even when the disclosure is negative.

Sample size	No sample-size calculation was performed. Behavioral and neural data reported in this study were highly over-powered; both are behavioral trial count and neural population size are larger than most neurophysiological studies in dorsal premotor cortex.
Data exclusions	Unless explicitly described, behavioral data without neural recordings are not reflected in the main body of the manuscript; we only report behavioral data during neural recordings. Regarding unit selection, units were kept if they were well-isolated and responsive to the task.
Replication	This project was carried out over two labs in different countries. We effectively replicated each other's results.
Randomization	Trials were randomly assigned in regards to their parameters (checkerboard difficulty, checkerboard dominant color, correct reach direction, stimulus-response association (color identity of the targets).
Blinding	No blinding was necessary for these experiments.

Reporting for specific materials, systems and methods

Materials & experimental systems

n/a	Involved in the study
☒	☐ Unique biological materials
☒	☐ Antibodies
☒	☐ Eukaryotic cell lines
☒	☐ Palaeontology
☐	☒ Animals and other organisms
☒	☐ Human research participants

Methods

n/a	Involved in the study
☒	☐ ChIP-seq
☒	☐ Flow cytometry
☒	☐ MRI-based neuroimaging

Animals and other organisms

Policy information about studies involving animals; ARRIVE guidelines recommended for reporting animal research

Laboratory animals	The study used two 9-year-old male Macaca mulatta monkeys.
Wild animals	The study did not use wild animals.
Field-collected samples	The study did not use field-collected samples.
